# Monkeypox: a global health emergency

**DOI:** 10.3389/fmicb.2023.1094794

**Published:** 2023-04-26

**Authors:** Ruxandra Ilinca Stilpeanu, Ana Maria Stercu, Andreea Lucia Stancu, Antoanela Tanca, Octavian Bucur

**Affiliations:** ^1^Victor Babes National Institute of Pathology, Bucharest, Romania; ^2^Faculty of Medicine, Carol Davila University of Medicine and Pharmacy, Bucharest, Romania; ^3^Brigham and Women’s Hospital and Harvard Medical School, Boston, MA, United States; ^4^Viron Molecular Medicine Institute, Boston, MA, United States; ^5^Genomics Research and Development Institute, Bucharest, Romania

**Keywords:** monkeypox, smallpox, zoonotic disease, ACAM2000, modified vaccinia Ankara, tecovirimat, mpox

## Abstract

Over the past 2 years, the world has faced the impactful Coronavirus Disease-2019 (COVID-19) pandemic, with a visible shift in economy, medicine, and beyond. As of recent times, the emergence of the monkeypox (mpox) virus infections and the growing number of infected cases have raised panic and fear among people, not only due to its resemblance to the now eradicated smallpox virus, but also because another potential pandemic could have catastrophic consequences, globally. However, studies of the smallpox virus performed in the past and wisdom gained from the COVID-19 pandemic are the two most helpful tools for humanity that can prevent major outbreaks of the mpox virus, thus warding off another pandemic. Because smallpox and mpox are part of the same virus genus, the Orthopoxvirus genus, the structure and pathogenesis, as well as the transmission of both these two viruses are highly similar. Because of these similarities, antivirals and vaccines approved and licensed in the past for the smallpox virus are effective and could successfully treat and prevent an mpox virus infection. This review discusses the main components that outline this current global health issue raised by the mpox virus, by presenting it as a whole, and integrating aspects such as its structure, pathogenesis, clinical aspects, prevention, and treatment options, and how this ongoing phenomenon is being globally approached.

## 1. Introduction

The first known human case of the zoonotic disease called monkeypox (mpox) was reported in 1970, although the virus that causes it was first identified in 1958, as a part of the Orthopoxvirus genus, in the Poxviridae family (Breman et al., 1980). A series of infections with poxviruses taking place from 1958 until 1968 started being observed in Denmark. This was observed in cynomolgus monkeys that had recently been brought from Singapore. The presence of a virus was confirmed after a thorough analysis of the samples taken from one of the monkeys that were showing a generalized vesiculopustular illness (Damon, 2011).

Further epidemiological investigations took place within that same period of time in the Democratic Republic of Congo, when an mpox-like virus was isolated for the first time in 1970 from a 9-month-old patient whose symptoms were suspected to be part of the smallpox clinical picture (Ladnyj et al., 1972). This case represented the beginning of acknowledging mpox as an ongoing endemic infection by the World Health Organization (WHO) within the African continent. This led to the observation of this health phenomenon in the central regions of the Democratic Republic of Congo, while also initiating certain animal studies (Parker and Buller, 2013), in the following decade, during the 1970s.

In this narrative review, we are summarizing the main clinical manifestations of the mpox disease, while also presenting the structure of the virus and including a classification of the main strains. Furthermore, we include a useful analysis on how this public health emergency is being handled globally, in terms of prevention, which is comprised the two vaccines recently approved by the US Food and Drugs Administration (FDA), ACAM2000 and JYNNEOS, and also in regard to the recommended treatment with antivirals, its main representant being tecovirimat. The latter is also known as ST-246 or TPOXX and it constitutes the first choice in treating mpox virus infections, due to the lack of major side effects, opposed to the other antivirals approved by the US FDA, called brincidofovir and cidofovir.

## 2. Structure of the orthopoxvirus genus, particularly monkeypox virus

Global research efforts were deployed once the cases of patients infected with an unknown member of the Poxviridae family were slowly increasing during the 1970s, a decade which was also marked by the cessation of smallpox vaccine production. Therefore, a vaccine which was once used to ensure cross-immunity to the mpox virus as well (Kennedy et al., 2009; Gilchuk et al., 2016; Kaler et al., 2022), was now stopped from being used. This allowed a more extensive group of patients to be at risk (Rimoin et al., 2010). However, the 2003 outbreak in the Western Hemisphere actually brought international awareness to the mpox virus. This was the first time a large number of cases outside Africa were confirmed, after native prairie dogs (Cynomys spp.) that had been in contact with African rodents were brought into the United States from Ghana (Reed et al., 2004).

**Figure 1:**
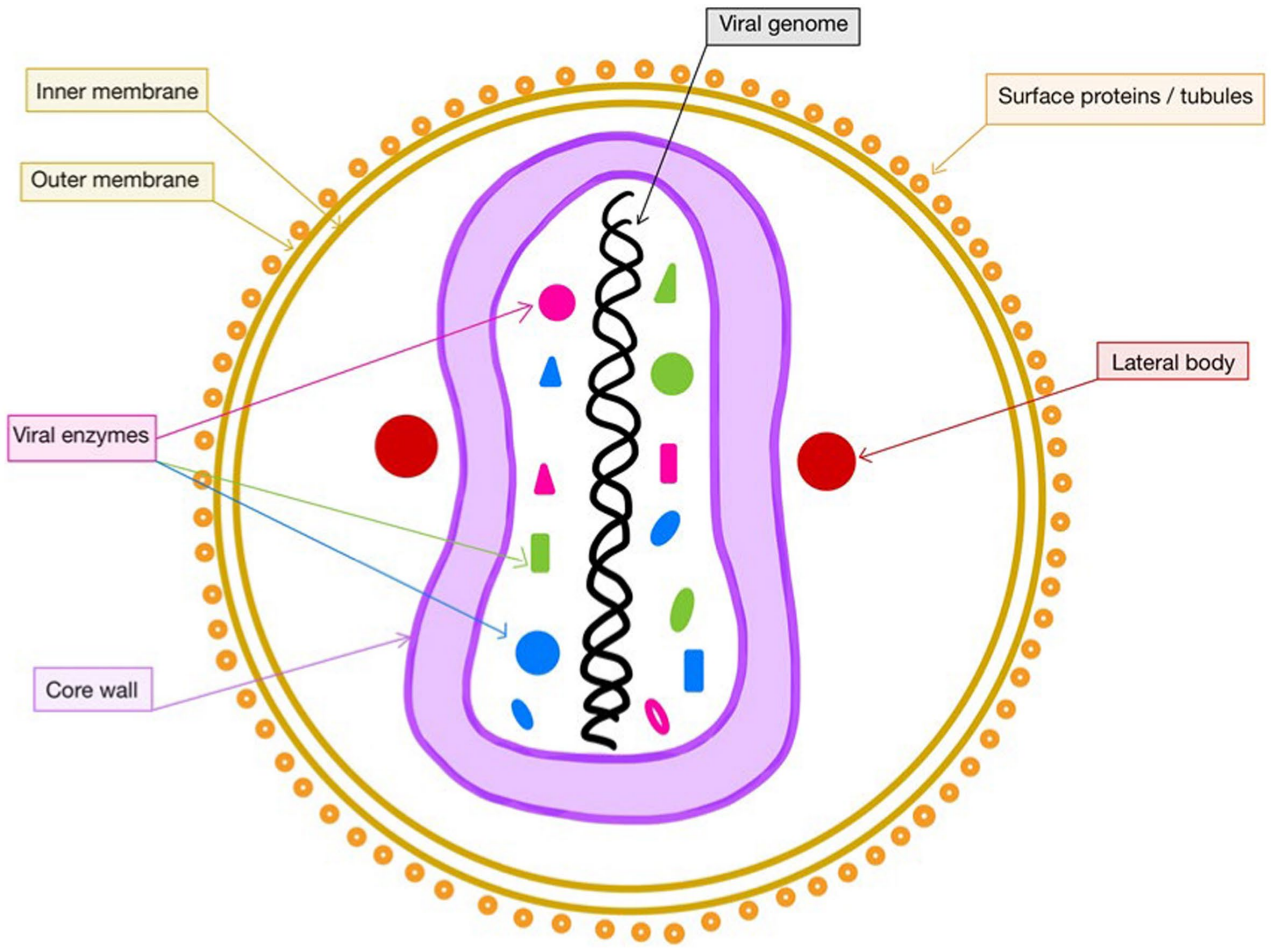
Structure of mpox. This figure was created with GoodNotes and adapted from Lu et al. (2022).

The general structure of poxviruses (Luna et al., 2022) was already known to be of brick-shaped particles, formed by large, linear, double-stranded DNA viruses that utilize virion proteins conserved in all species, which allow them to replicate in the cytoplasm (Moss, 2013; Figure 1). However, it was outlined that due to its large size, the mpox virus barely crosses the host’s natural barriers used for protection against infections. Consequently, it needs a set of rearranged viral proteins that would enhance its resistance against the host’s foreseeable immune responses (Kaler et al., 2022).

The intracellular proteins that help modulate the actions of the mpox virus are first represented by the virotransducer ones, which interact with the apoptotic pathways. Secondly, the virostealth proteins are involved in the process of downregulation of immune recognition molecules, such as major histocompatibility complex class I and CD4. Thus, they lower the chance of the virus being identified by the immune system. The extracellular viral modulators, also known as viromimic proteins, consist of viroreceptors, reducing the host’s cytokines and chemokines impact and virokines, which are in a quest to imitate the host’s cytokines and chemokines (Okyay et al., 2022). Under the conditions of not having a fully developed immune system, young patients are prevalent among cases with poxvirus infection, due to the lack of maternal antibodies (Hurisa et al., 2019), followed by immunodeficient patients that are not capable of ensuring a competent immune response.

Although the mpox virus shares its linear double-stranded DNA genome with variola and vaccinia viruses (di Giulio and Eckburg, 2004), the former does not inherit all of the properties that the smallpox virus holds, such as the lack of open reading frames (ORFs) in the inverted terminal repeat (ITR) region, which contains the origins of replication (Hurisa et al., 2019). However, this ORF comparison further led to the mpox virus’ ability to interact with the complement, in both its classical and alternate pathways. In comparison, one of the other members of the same genus, the variola virus, which specifically targets human hosts, also manages to efficiently inhibit the human complement system through its proteins, which are better designed to defeat the innate immune response (Rosengard et al., 2002).

Another particularity that differentiates the mpox virus structure from the rest of the orthopoxviruses is given by the rather incomplete inhibition of E3L and K3L orthologs, derived from genes of the vaccinia virus. These mutations are considered to be the source of attenuation of mpox virus in a wide range of hosts, thus improving its transmission rates (Haller et al., 2014).

## 3. Pathogenesis of the monkeypox virus

So far, two distinct clades of mpox virus have been declared, represented by the African regions with their highest prevalence (Samson Enitan, 2022). The Central African category, also called the “Congo Basin mpox virus clade” or clade I, could be considered more virulent than the West African (clades IIa and IIb) one due to its increased effectiveness in blocking the complement-initiated viral neutralization. Regarding the molecular aspect, the latter strain proved to have an additional 453-nucleotide residue, during a LightCycler quantitative PCR analysis (Saijo et al., 2008), which was used for the diagnosis of this disease, by targeting the A-type inclusion body gene of the mpox virus.

Further epidemiological studies revealed a parallel presence of antibodies among unvaccinated patients in both the Western and Central regions, although the Congo area held more than 90% of these cases, including the only fatal ones. The Central African mpox presents a greater rate of morbidity and human-to-human transmission, also confirmed by the 2003 US outbreak, which had no case-fatalities, since the initial strain was West African (Chen et al., 2005).

The mechanisms behind this high virulence reside in a suppression of the inflammatory cytokine production in human cells. This phenomenon is caused by preventing T-cell activation (Zandi et al., 2023a) and by hindering apoptosis processes in the infected host (Wilson et al., 2014). Moreover, the West African strain presents a lack of complement enzyme‘s inhibition. Thus, it manages to become an important immune-modulating factor that contributes to the enhanced viral load of the Central African strains.

This peculiarity of the virus being able to suppress the host’s T-cell response (Hammarlund et al., 2008), which further outlines its antibody-dependent neutralization, also noticed in vaccinia’s case (Isaacs et al., 1992), could explain why the Congo clade was associated, in some cases, with longer periods in which the virus was detected in the patients’ blood samples (Likos et al., 2005).

## 4. Transmission of the monkeypox virus

Although it was first discovered in monkeys, mpox virus does not originate in these mammals (Kaler et al., 2022) and has a wider range of possible hosts, due to its better transmissibility made possible through the aforementioned attainment of E3L and K3L orthologs, after recombining with the rest of the Poxviridae family members.

Although the main host reservoir of mpox is still unknown, small animals, such as rope squirrels (Funisciurus sp.), sun squirrels (Heliosciurus sp.), and Gambian giant rats (Cricetomys sp.; Haller et al., 2014), could act as the host reservoirs of the mpox virus. In contrast to this broad availability, variola virus proved to be human-specific since its beginnings, while mpox only started being considered a health threat for humans after the discontinuance of mass vaccination against the once devastating smallpox disease (Essbauer et al., 2010).

Being an emergent zoonotic disease, the usual source of infection for humans with mpox virus is direct exposure to infected animals (Nolen et al., 2015; Quiner et al., 2017).

Regarding human-to-human transmission, which is currently considered to be extensive (Nolen et al., 2016), individuals get infected through getting in touch with mpox virus patients’ mucocutaneous lesions or their respiratory droplets, given the presence of the virus in their oropharyngeal secretions. Even though the efficiency of this interhuman transmission is lower than the one in the case of variola virus, it did appear in almost 11.7% of household contacts of several patients that were non-vaccinated with the smallpox vaccine, which was known to be protective against mpox (Saijo et al., 2009).

**Figure 2:**
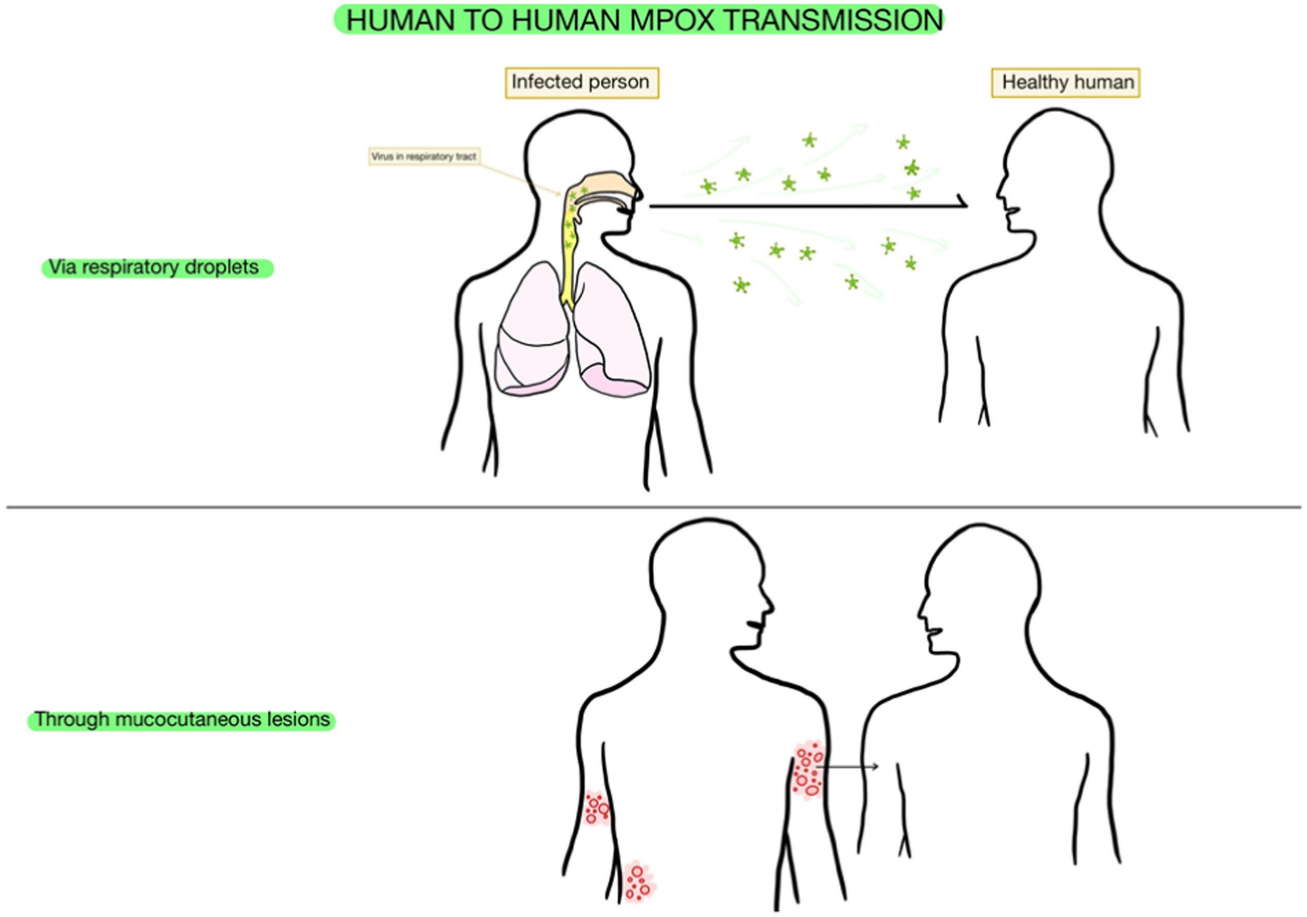
Transmission of mpox. This figure was created with GoodNotes and adapted from Wang et al. (2021).

Therefore, both salivary and airborne ways of transmission are significant and should present awareness among healthcare workers (Yang et al., 2023). Moreover, mpox testing can rely on saliva and air samples as materials for diagnosis (Allan-Blitz et al., 2023; Figure 2).

## 5. The clinical picture of the disease produced by a monkeypox virus infection

During a study of the two strains of mpox virus in non-human primates, the viral load levels in the monkeys infected with the Congo Basin one (clade I) proved to be almost 10 times higher than the ones with the Western African (clades IIa and IIb) mpox variation. Moreover, the more virulent clade also proved to spread into the respiratory, genito-urinary and digestive systems more harshly than the other one, which could be considered milder in terms of manifestation (Damon, 2011).

What should be noted is that the clinical picture of the mpox virus consists of symptoms which are characteristic of smallpox (Sale et al., 2006), such as the initial fever of the later onset, headache, and fatigue. However, a major difference resides in the presence of lymphadenopathy among mpox virus patients, which could possibly indicate a more effective immune response given by the host in this case (Wilson et al., 2014).

Regarding the incubation period, two intervals were outlined during the 1980s mpox virus observations. One of them, which was approximately 10–14 days, the asymptomatic time frame, was measured from the exposure moment until the fever onset. The other one was estimated to last until the rash onset, and it was considered to take about 12–16 days. However, the period that lasts from the fever episodes until the rash burst also proved to be longer in the case of non-vaccinated patients (Damon, 2011).

The rash burst marks the moment in which the fever level lowers. The lesions begin by appearing slightly confluent, but can evolve into specific maculopapular and vesiculopustular phases, which could be a differentiating criterion from other diseases that present the same skin manifestation (Jezek et al., 1986). Varicella, an illness caused by the varicella zoster virus, also causes a rash as a symptom. However, in comparison with mpox and smallpox, it progresses faster, and the manifested fever is milder (Wilson et al., 2014).

Separating the mpox virus diagnosis from the variola virus one can only be performed through laboratory extended analysis (Weaver and Isaacs, 2008). Other lesions may appear in the oropharynx in most unvaccinated patients, such as oral ulcers, tonsillitis, or cough (Joseph and Anil, 2022). Ocular symptoms, such as conjunctivitis and blepharitis, may occur as well (Damon, 2011).

Perhaps, the sign with the highest incidence among mpox virus patients is represented by lymphadenopathies that appear in the cervical and submaxillary areas (Nalca et al., 2005). They usually occur early on in the evolution of the disease, about 1 or 2 days after the fever episode ends. The size of the inflamed lymph nodes ranges between 1 and 4 cm in diameter, with a firm consistency and a possible appearance of pain during their examination.

One difficulty arises in the process of diagnosing patients with mpox who already have the human immunodeficiency virus infection and syphilis, due to the fact that they can present with atypical clinical aspects. A thorough screening for mpox should be initiated among patients with skin affections, therefore decreasing the risk of spreading mpox in hospitals (Jang et al., 2023).

Overall, mpox cases last approximately 2–4 weeks (Weaver and Isaacs, 2008), from the data gathered so far, even despite possible complications. Although there have been mpox cases with complications due to possible secondary infections from bacteria, such as pulmonary and digestive ones, with symptoms such as diarrhea or vomiting, most of the patients heal within 2–4 weeks. Moreover, the only possible consequences that appeared post-illness in some survivors were major scarring of the skin and blindness (Parker et al., 2012; Brown and Leggat, 2016; Kaler et al., 2022).

To sum up the classification mentioned before, mpox is considered to have the following major clades: clade I, or the Congo strain; clade IIa, which exists in West Africa and presents low mortality; and clade IIb, which is currently being spread worldwide through human-to-human transmission (Americo et al., 2023).

In a long-term follow-up study that included clade IIb mpox patients, the residual morbidity after they were healed was studied. During their mpox infections, they presented with nonspecific prodromal symptoms and localized lesions of the skin and mucosae, surrounding the anal and genital regions. Two-thirds of them still had anorectal pain and genital issues at their follow-up, 3–20 weeks after healing, besides persistent fatigability, which was also seen in other infectious diseases such as COVID-19 (White et al., 2001). The conclusions of the study were that most of the patients still had ongoing symptoms weeks after their initial disease disappeared. Therefore, physicians should be conscious of the pain and possible issues that still affect the mental health of their patients, after an apparently resolved disease, such as the infection with the clade IIb mpox strain (Berens-Riha et al., 2023).

## 6. The current global situation regarding outbreaks of monkeypox virus infections

For the past 2 years, the world has faced the impactful COVID-19 pandemic, with tremendous impact upon so many aspects of our lives, such as health, economy, and tourism (Doherty, 2021; Fortner and Schumacher, 2021; Haq et al., 2021; Rabbi et al., 2021; Walia et al., 2021). As the COVID-19 pandemic situation globally ameliorates, the fear of a new epidemic phenomenon happening becomes more and more prevalent, as mpox virus cases arise in areas beyond the endemic ones in Africa, spreading in European countries and in the Western Hemisphere (Kaler et al., 2022).

Given this situation, considered to be a multi-country issue of high risk, the Director-General of WHO declared the ongoing mpox outbreak as a public health event of international concern (WHO, 2022a).

Since the beginning of the year 2022 and as of 22 August 2022, 41,664 mpox cases and 12 fatalities were confirmed in about 96 countries, in all the six WHO regions, in an epidemiological update from WHO regarding the multi-country ongoing outbreak of this disease (WHO, 2022b). The report revealed that 23 countries presented an increasing number of cases compared to the previously declared situation in the last WHO report, with the highest rise in the United States of America. The Democratic Republic of Congo remains the country most affected by the mpox virus, with continuous reports over the last five decades regarding confirmed cases (Bunge et al., 2022). After the disease was no longer seen as endemic, but a worldwide outbreak, it was declared as an emergency independent of any travel causal factors, but with a major negative contribution brought by sexual transmission (Karagoz et al., 2023).

Further epidemiological investigations confirmed the fact that most of the affected patients, in a proportion of 95.8% of the cases reported with sexual orientation to the WHO, are represented by men aged 20–50, who identify themselves as men having sex with men (MSM), without any recent journeys to mpox endemic countries from Africa (Samson Enitan, 2022). However, there were multiple cases among heterosexual patients as well, this being a reason which opposes the stigmatization of the MSM community. What is more, a bigger threat is represented by countries where hygiene is not up to the accepted standards, this matter should be addressed first, since the mpox DNA is also found in urine and faeces (Singla and Shen, 2022).

Due to the increasing number of infected cases in their country, Portuguese scientists managed to release the first draft of the genome sequence of the mpox virus strain that had been recently identified in the non-African outbreak. After gathering genetic data, it was proved that the 2022 mpox virus is part of the West African mpox virus clade (Simpson et al., 2020).

The clinical management suggested by the WHO represents an advisory for Member States to pursue their national immunization technical advisory groups and develop recommendations regarding immunization for mpox, for groups of high risk in their countries (WHO, 2022b).

Immunocompromised people, pregnant women, and young people present a more prevalent risk of exposure. The recently approved vaccines for prevention of mpox virus are JYNNEOS and ACAM2000, as mentioned by the US Centers for Disease Control and Prevention (CDC; CDC, 2022a). The latter vaccine represents an alternative to the former one, given the fact that it only has a single dose. However, it also comes with more impactful, apparent potential side effects.

Regarding potential therapeutics, on May 2019, 2022, United States, Canada, and Europe confirmed that tecovirimat (Tpoxx) can be used for means of treatment of both patients with smallpox and patients with mpox (Otu et al., 2022).

Another issue of important matter in facing the mpox phenomenon resides in misinformation of people, causing a so-called “misinfodemic” (Brainard and Hunter, 2020), with many false claims, such as those linking mpox to COVID-19 vaccination, especially the AstraZeneca vaccine and also the fact that many social media platforms spread the idea that the images with the clinical manifestations such as skin rashes of the mpox are nothing more than re-edited old photos from previous African outbreaks. These altogether manage to induce a lack of trust from readers that want to get informed on the current mpox situation, through making the untrustworthy sources hard to be differentiated from the reliable ones (Ennab et al., 2022).

Although measures are being globally taken, most challenges occur in the areas which are perhaps most in need, with the highest numbers of cases. People from African endemic regions have to deal with issues such as the expenses that arise from the use of healthcare facilities and limit their intentions of accessing them, further leading to underreporting of cases, with an impact on worldwide surveillance. Furthermore, there is also a limited availability of vaccine provisions and therapeutics due to the low possibility of African countries producing their own supplies (Samson Enitan, 2022).

## 7. Measures of prevention against potential pandemic outbreaks

Public health awareness from all over the world could be improved and coordinated efforts on an international level should be made in order to take proactive measures regarding the prevention of even more cases of mpox than the current situation, thus not allowing it to become the next pandemic (WHO, 2022c).

Information sharing among countries worldwide, disease control via personal and collective hygiene, and monitoring new cases are important rules when it comes to global prevention. It is especially important to start all these measures in the early stages of an infection, and immediate action is necessary for controlling further outbreaks. Another effective measure is vaccination. The US FDA approved two vaccines that may provide protection against mpox disease, ACAM2000 and JYNNEOS (Poland et al., 2022). These vaccines are approved for certain individuals that are at high risk for contacting this virus, as well as for the relatives and direct contacts of those infected with mpox virus (Luo and Han, 2022).

It is of high importance that groups of individuals at risk, such as men having sex with men (Martínez et al., 2022), are informed and have access to knowledge campaigns. Raising awareness among these groups of people is especially crucial, but the remaining population should not be neglected, as any individual should benefit from medical prevention education. Groups at risk should be a priority (Harapan et al., 2020) and they should be the first category taken into consideration when it comes to campaigns, sharing information and raising awareness efforts.

The result of vaccination is an improved clinical response to the infection, milder symptoms and faster recovery (Zandi et al., 2023b). The best and most effective measure of pre-exposure to the virus prophylaxis is vaccination (Rizk et al., 2022). Alongside vaccination, another useful measure of global prevention is isolation of the individuals with this infection, restricting their contact with other humans (CDC, 2022b). Because the incubation time of the virus is well known, close contacts of those infected should be traced and reported to the health department as fast as possible, accompanied by meticulous monitoring (Meaney-Delman et al., 2022).

## 8. Vaccines approved for smallpox, monkeypox, and other orthopoxviruses

Since smallpox was eradicated, the most common Orthopoxvirus infection in humans known today is the infection with the mpox virus (Sklenovská and van Ranst, 2018). As mentioned before, due to its antigenic similarities to the smallpox virus, the vaccines developed for the smallpox disease have cross-protection against the mpox virus and against other Orthopoxviruses (Poland et al., 2022). Because the smallpox vaccine brought about the eradication of this pathology, more than 70% of the global population today has never received the smallpox vaccine. Therefore, the global population is very susceptible to an Orthopoxvirus infection (Kmiec and Kirchhoff, 2022).

Currently, there is a low immunity (Shafaati and Zandi, 2022, 2023) in the population that was vaccinated prior to the eradication of the smallpox virus, in 1980 (Reynolds and Damon, 2012). Recent data suggests that this vaccination taken place over 25 years ago may still offer at least some protection to these individuals against an infection with an Orthopoxvirus (Wilson et al., 2014).

At the moment, there are only three available vaccines against mpox: ACAM2000, JYNNEOS, and LC-16, the first one being a second-generation vaccine and the latter two being third-generation vaccines.

LC-16 contains the Lister strain of Vaccinia and is partially replicating as it is an attenuated strain. It can be administered in a single dose. It is a vaccine that lacks severe adverse reactions, and the local and systemic adverse events are mild and easily manageable (Kenner et al., 2006; Saito et al., 2009; Nishiyama et al., 2015). The vaccine used in the past for the eradication of smallpox (Dryvax—ACAM2000), a live vaccine incorporated with unattenuated vaccinia virus strains, is highly dangerous, as it can generate alarming side effects, leading to long-term sequelae (Zielinski et al., 2010). This vaccine is considered to be the gold standard for its efficacy, because it is highly immunogenic. However, it preserves enough residual virulence in order to produce threatening aftereffects and this residue is transmissible, as well. Recent studies have reported that there is a persistence of both cellular and humoral immunity after a single dose of Dryvax. However, as mentioned before, it is highly unsafe (Zielinski et al., 2010; Moss, 2013). This vaccine is not in use for the mpox infection, since it is not approved by the US FDA. Since there is a large number of high-risk consequences, there is a need for a safer vaccine that can match the effectiveness of the original vaccine (Zielinski et al., 2010). Currently, there are several approved vaccines for the smallpox virus (Wilson et al., 2014), as presented in Table 1.

**Table 1:** FDA approved vaccines for the smallpox virus.

Vaccine	Type of vaccine	Pros	Cons and side effects	Stage of development or use	References
ACAM2000	Live vaccinia virus/replication competent vaccinia virus (a derivative of the original Dryvax)	-Single dose-long-term storage.	Lesion at vaccination site -replication in mammalian cells is transmissible-cardiac complications post-vaccination were reported -contraindicated in immunosuppressed individuals, atopic patients, patients with cardiac diseases and pregnant women-high risk of inadvertent inoculation and autoinoculation, which may lead to uncontrolled viral replication-similar reactogenicity and residual virulence profile as Dryvax.	Licensed in the US.	Zielinski et al. (2010); Rizk et al. (2022)
Modified vaccinia Ankara (MVA)/Imvamune/Imvanex/JYNNEOS	Attenuated vaccinia virus/replication-deficient modified vaccinia Ankara	-Does not achieve complete replication in mammalian cells—protection was reported in primate models injected with lethal doses of mpox virus—no lesion at injection site-can be administered to immune suppressed individuals-can be used as a pre-vaccine -stimulates antibody production in atopic patients as well as in immunosuppressed individuals.	-Two doses, 4 weeks apart	Authorized by European Commission. Licensed by US FDA in 2019. Indicated for prevention of an orthopoxvirus infection, such as mpox.	Earl et al. (2004); Moore et al. (2022)
LC16m8	Attenuated vaccinia virus	-Single dose-less adverse reactions-prevents viral replication-protection was reported in non-human primates against severe mpox disease.	-Replication of the attenuated virus in mammalian cells.	Licensed in Japan. More than 50,000 schoolchildren vaccinated.	Kennedy et al. (2009)
Aventis pasteur smallpox vaccine (APSV)	Replication-competent live vaccinia virus		-Contraindicated in immunosuppressed individuals.		Islam et al. (2022)

The only vaccine approved by the US FDA, JYNNEOS, is also used for the prevention of smallpox, since it is based on a harmless vaccinia virus, with a decreased viral load (FDA, 2022). It is a replication-deficient live virus vaccine that contains a weakened Orthopoxvirus virus, the Modified Vaccinia Ankara-Bavarian Nordic virus (Sah et al., 2023). This vaccine was originally created as a weapon against bioterrorist attacks by smallpox virus. This vaccine is safer than the ACAM2000 (Sah et al., 2023) and the upper hand of this vaccine is that it is also considered safe for immunocompromised patients who may not always be allowed to get vaccinated with live vaccines (Overton et al., 2015). JYNNEOS appeared as a replacement for the ACAM2000 vaccine, which had multiple adverse reactions, the former one being authorized for emergency use, as well (Rao et al., 2022). The adverse reactions of this vaccine are much less severe and much more manageable than those of the ACAM2000 vaccine. In contrast to the administration of the JYNNEOS vaccine in two-dose series, ACAM2000 consists in only one dose which forms a vaccination site, outlined by multiple punctured spots. This leads to its adverse properties being spread to other areas of the body, an infection of the eye with the vaccinia virus being a valid possibility, eventually leading to blindness. Through the numerous adverse reactions of the ACAM2000 vaccine, myocarditis and pericarditis, but also swelling in the central and peripheral nervous systems, occur most often (Faix et al., 2020). On the other hand, in the JYNNEOS vaccine, severe adverse reactions that were observed after ACAM2000 vaccination, such as myocarditis and pericarditis, were not reported, turning this vaccine into a much safer option for the population (Yang and Yonts, 2019). Because of the need of a safer vaccine, one that can match the efficacy of the first-generation vaccines, scientists reported that integrating the human interleukin-15 cytokine into the genome of the Wyeth strain of vaccinia (Wyeth/IL-15) results in a vaccine with higher immunogenicity in mouse models. IL-15 is a cytokine with multiple effects on both innate and adaptive immunity, being necessary for the development, differentiation, activation, and proliferation of CD8+ T-cells, dendritic cells, and natural killer (NK) cells. These cells, especially NK cells, play a central role in the clearance of the virus (Zielinski et al., 2010). Taking these into consideration, the Wyeth/IL-15 vaccine may be a better alternative for the modern population, as it may be safer and highly effective.

## 9. Antiviral drugs approved for the treatment of monkeypox infections

**Figure 3:**
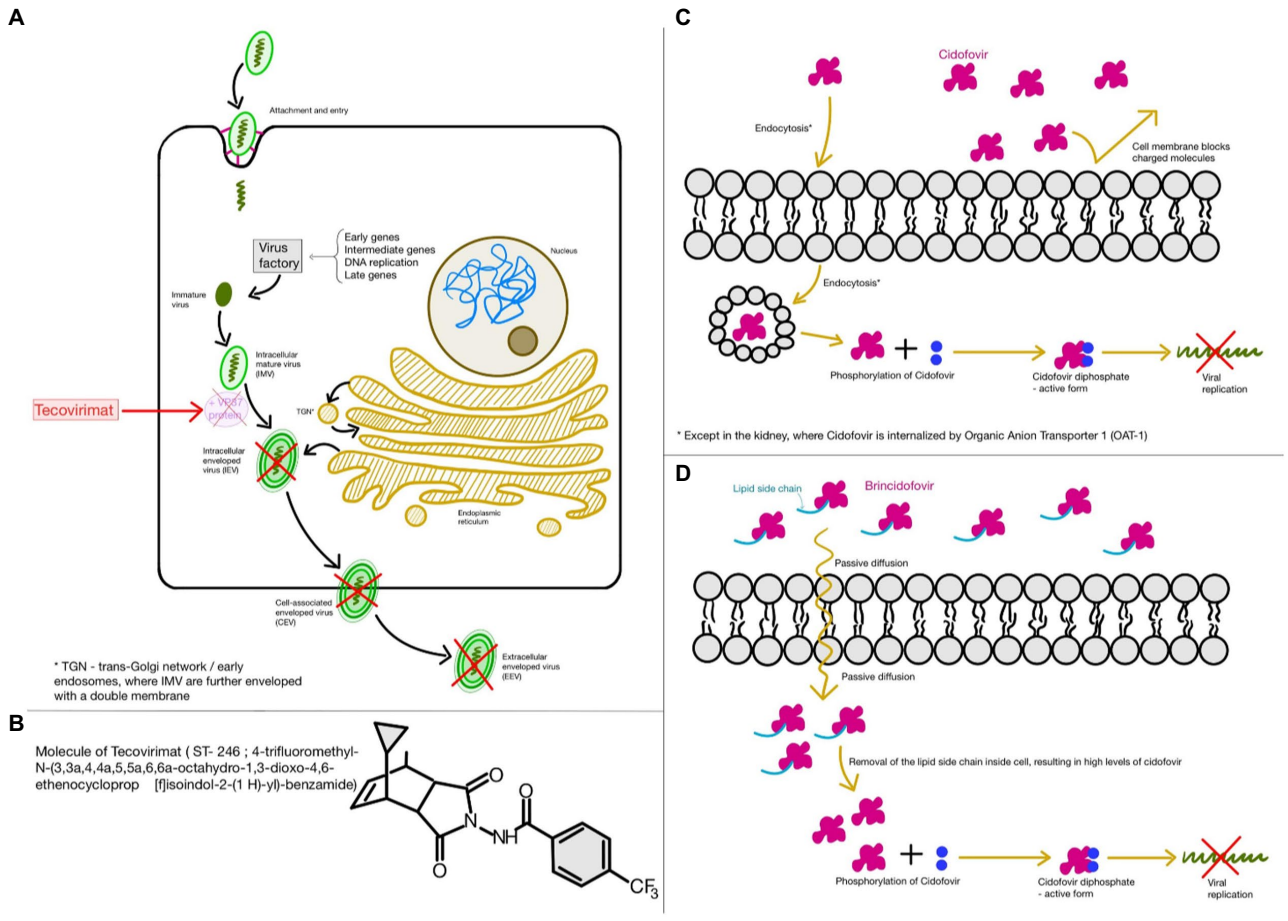
Food and Drugs Administration (FDA) approved drugs for the treatment of both smallpox and monkeypox virus infections **(A,B)**; Tecovirimat; **(C)** Cidofovir; **(D)** Brincidofovir. This figure was created with GoodNotes.

Since there is no approved drug and current specific treatment for the mpox virus infection and because there is a high structural resemblance between the mpox virus and the smallpox virus, both being part of the Poxviridae family, Orthopoxvirus genus, antiviral drugs used, and approved for the infection with smallpox virus may potentially be used in treating mpox virus infections as well. The main purpose of the clinical treatment is to improve the quality of life by reducing the chances of developing long-term sequelae. The drugs approved by the US FDA are tecovirimat, brincidofovir, and cidofovir in adults and children (Russo et al., 2020b; Luo and Han, 2022; Figure 3). These drugs have already been approved for the treatment of the infections with the smallpox virus. Thus, they could potentially be used for the treatment of the mpox virus infection.

Out of these three antiviral drugs, tecovirimat, also known as ST-246 or TPOXX, is of first choice, since it does not present significant side effects and unveils a remarkable reduction in viremia. Its mechanism of action consists of inhibiting the virus replication *in vitro*, by targeting the variola virus VP37 protein related to the release of enveloped virions (Jordan et al., 2009; Russo et al., 2018). Although this drug does not prevent viral replication, it is very effective against viral dissemination (Russo et al., 2020a). It has been observed that, at low concentrations, this antiviral drug is capable of inhibiting the cytopathic effect of poxviruses. However, in order to inhibit other RNA and DNA viruses, a higher concentration is required (Stafford et al., 2023). US CDC offers the latest recommendations, detailed guidelines, and a protocol for the access and use of tecovirimat in mpox virus infections and other non-variola orthopoxvirus infections, in both adults and children (Centers for Disease Control and Prevention Center for Preparedness and Response Monkeypox Outbreak, 2022). The recovery after treatment with tecovirimat is faster than with the other two drugs. Tecovirimat has good oral bioavailability and has one important adverse reaction reported, pulmonary embolus. Other adverse reactions are more common, and they include headache, dizziness, nausea, and vomiting (Stafford et al., 2023).

Cidofovir, a nucleotidic analog and an antiviral drug that acts by inhibiting viral DNA polymerase, is known to exhibit nephrotoxicity. This is a prodrug and its active form, cidofovir diphosphate is obtained after intracellular phosphorylation. Because of its toxic effects on the kidneys, it is usually administered alongside oral probenecid and pre-hydration fluids. Unlike tecovirimat, it has poor oral bioavailability. Therefore, it is intravenously administered (Stafford et al., 2023).

However, CMX-001 is a modified cidofovir compound that has no adverse reactions and complications toward the kidneys and showed promising results against a variety of Orthopoxvirus species (MacNeil et al., 2009).

Brincidofovir is the oral analog of the intravenous cidofovir, and it may be safer, since it is less toxic upon the renal system. However, there have been reports suggesting that brincidofovir is highly toxic for certain human organs, being associated with liver malfunction, since it may increase transaminases and bilirubin in the serum (Quarleri et al., 2022; Rizk et al., 2022). On the other hand, in case of a severe mpox disease, tecovirimat in association with brincidofovir is indicated by some studies (Rizk et al., 2022).

Furthermore, an adjuvant to these drugs would be the vaccinia immune globulin, which is a hyperimmune globulin that could be very useful in the treatment of specific complications and adverse reactions regarding the vaccinia vaccination, as licensed by the US FDA. Although it sounds promising as a possible treatment, the lack of data regarding its effectiveness against mpox and the absence of human testing shape the image of this drug into one that needs to be further investigated, although clinical trials completed so far rarely indicate any serious adverse reactions and no discontinuation of the vaccinia immune globulin took place (Lai et al., 2022; Rizk et al., 2022).

Reported data have shown that antiviral treatment (tecovirimat), either used alone or as an adjuvant to vaccination, is fully protective against an mpox infection, as opposed to vaccination alone. Moreover, antiviral drugs do not interfere with the protective immunity induced by vaccination, by not compromising it, as shown in both mice and monkeys (Berhanu et al., 2015).

It is of high importance to know how antiviral drugs and vaccines interact in order to trigger the best immune response in individuals. As mentioned before, tecovirimat is of first choice when it comes to antiviral therapy. Thus, the interaction of this drug with vaccines was intensely studied (Desai et al., 2022).

Tecovirimat has exhibited full protection against mpox disease when administered up to 72 h post infection in cynomolgus macaques, 5 days, post infection, intravenously, in non-human primates and up to 14 days post infection, *via* aerosol challenge, in these non-human primates. All in all, 100% survival was reported when this drug was administered before the emergence of clinical symptoms (Russo et al., 2020a).

Studies on animals have reported that, when treated with tecovirimat, post-administration of the ACAM2000 vaccination site lesions was milder and the adverse reactions were lighter, as well. These studies were done on both vaccinated and non-vaccinated animals, to investigate the behavior of the vaccine-antiviral association. Whether the animals were vaccinated or not, when treated with tecovirimat/TPOXX, they survived, suggesting that this drug does not interfere with the effects of the vaccine. The results of animal testing imply that the combination tecovirimat-vaccine attenuates the reactogenicity (Russo et al., 2020a). While the protective efficacy of the vaccine, when measured by survival, is not affected by tecovirimat, the humoral response to the vaccine may be diminished, resulting in a lower efficacy of the vaccine. In comparison, tecovirimat is highly effective even in infected patients who present a progression of their clinical symptoms, while the efficacy of the vaccine decreases with the advance of the disease and it should be administered as close as possible to the first manifestations of the disease (Russo et al., 2020a). The administration of the vaccine post-exposure to mpox virus does not provide a protection. However, post-exposure treatment with tecovirimat, with or without vaccination, offered full protection (Berhanu et al., 2015).

## 10. Discussion and conclusion

Although the world currently faces a potential epidemic of the mpox virus, past knowledge and research performed on the Orthopoxvirus genus is highly advantageous in the present days. The existence of potent vaccines and effective antiviral drugs against mpox virus are a few of the measures that can easily prevent major outbreaks. Therefore, vaccines such as ACAM2000 and JYNNEOS, also known as Modified Vaccinia Ankara (MVA), are useful against mpox for both infection prevention and treatment. In addition, the use of the tecovirimat antiviral drug is highly effective against an infection with the mpox virus. The vaccinia immune globulin, a hyperimmune globulin, could be used as an adjuvant to antivirals. This hyperimmune globulin could prevent, or even treat, if there is the case, complications and adverse reactions to the vaccines. However, further studies are required, because there are not enough data and research about this globulin and its use as a vaccine could be considered unsafe so far.

Moreover, the wisdom (Zhu et al., 2020) and awareness and knowledge (Elie, 2020; Jan et al., 2020; Mouffak et al., 2021) that the world has gained since the COVID-19 pandemic (Habas et al., 2020; Saha et al., 2020) are of extreme use in avoiding catastrophic outcomes with the monkeypox virus. By using everything that is known up to the present moment, such as the acknowledgments of the Orthopoxvirus genus, the research and clinical trials on both vaccines and antiviral drugs, as well as the insights from the most recent pandemic, the COVID-19 pandemic, another potential pandemic with the mpox virus could be avoided with ease. “History repeats itself” is a very true, well-known saying. Thus, it is an opportunity for humans to learn from the past, in order to avoid making the same mistakes in the future.

## Author contributions

RIS, AMS, and OB designed and organized the review. RIS and AMS analyzed and summarized the information, and wrote the manuscript. OB, ALS, and AT supervised the work and contributed to the writing and improvement of the manuscript. All authors contributed to the article and approved the submitted version.

## Funding

ALS was funded by a T32 grant from the US NIH. OB is funded by a grant of the Romanian Ministry of Education and Research, CNCS-UEFISCDI, project number PN-III-P4-ID-PCE-2020-2027, within PNCDI III. The authors would like to acknowledge the funding from the Ministry of Research, Innovation, and Digitization in Romania, under Program 1—The Improvement of the National System of Research and Development, Subprogram 1.2—Institutional Excellence—Projects of Excellence Funding in RDI, Contract No. 31PFE/30.12.2021.

## Conflict of interest

The authors declare that the manuscript was developed in the absence of any commercial or financial relationships that could be construed as a potential conflict of interest.

## Publisher’s note

All claims expressed in this article are solely those of the authors and do not necessarily represent those of their affiliated organizations, or those of the publisher, the editors and the reviewers. Any product that may be evaluated in this article, or claim that may be made by its manufacturer, is not guaranteed or endorsed by the publisher.
